# Characterization of Conductive 3D Printed Fingertips Manufactured by Fused Filament Fabrication

**DOI:** 10.3390/polym15061426

**Published:** 2023-03-13

**Authors:** Zhao Kai, Imjoo Jung, Sunhee Lee

**Affiliations:** 1 Department of Fashion Design, Changzhou Vocational Institute of Textile and Garment, Changzhou 213164, China; 2 Department of Fashion and Textiles, Dong-A University, Busan 49315, Republic of Korea; 3 Department of Fashion Design, Dong-A University, Busan 49315, Republic of Korea

**Keywords:** 3D printed fingertips, infill density, infill pattern, compressive property, electrical property

## Abstract

This study purposed to develop conductivity 3D printed (3DP) fingertips and confirm their potential for use in a pressure sensor. Index fingertips were 3D printed using thermoplastic polyurethane filament with three types of infill patterns (Zigzag (ZG), Triangles (TR), Honeycomb (HN)) and densities (20%, 50%, 80%). Hence, the 3DP index fingertip was dip-coated with 8 wt% graphene/waterborne polyurethane composite solution. The coated 3DP index fingertips were analyzed by appearance property, weight changes, compressive property, and electrical property. As results, the weight increased from 1.8 g to 2.9 g as infill density increased. By infill pattern, ZG was the largest, and the pick-up rate decreased from 18.9% for 20% infill density to 4.5% for 80% infill density. Compressive properties were confirmed. Compressive strength increased as infill density increased. In addition, the compressive strength after coating was improved more than 1000 times. Especially, TR had excellent compressive toughness as 13.9 J for 20%, 17.2 J for 50%, and 27.9 J for 80%. In the case of electrical properties, the current become excellent at 20% infill density. By infill patterns at 20% infill density, TR has 0.22 mA as the best conductivity. Therefore, we confirmed the conductivity of 3DP fingertips, and the infill pattern of TR at 20% was most suitable.

## 1. Introduction

Recent developments in wearable robotic exoskeletons have great potential in addressing these issues through automating rehabilitation therapy and providing physical assistance for patients [1,2]. The devices need to focus on two main features, namely customizability to fit different hand dimensions and portability to make it usable in performing daily tasks outside the hospital especially as assistive device. This evolution is the result of a desire for lighter, more comfortable gloves, along with a desire for a greater design flexibility. In particular, it is very important for the exoskeleton hand to apply soft materials to act like human hands [3]. These days, many studies use 3D printing for personalized manufacturing with soft materials. Heung et al. (2019) reported 3D printed soft robotic hand ‘SECA (Soft-Elastic Composite Actuator)’ for stroke rehabilitation using shore 30 A elastomer with bending actuator. The clinical effectiveness of the SECA was evaluated [4]. Mohammadi et al. (2018) investigated a 3D printed soft exoskeleton robotic ‘Flexo-glove’ for impaired hand rehabilitation and assistance using Shore 90 A thermoplastic polyurethane (TPU), attaching the actuator to part of the wrist. Then, performance evaluation of Flexo-glove was performed with three grasping motions, i.e., cylindrical, spherical, and pinch [5].

To automatically detect the person’s movements, it is very important to provide distributed touch and haptic feedback. This can be seen in soft robotics, electrode skin (E-skin), artificial muscles, sensors, actuators, computing devices, and so on. This can be accomplished by applying a pressure sensor. A pressure sensor can be used to follow the tactile sensation of humans. For developing a pressure sensor applied to robotic hand, it needs to be flexible, soft, stretchable, and possess conductivity [6,7,8]. In order to achieve good performance, a stretchable polymer with conductive fillers can be used [9,10,11,12]. In addition, in order to apply the sensor to the body or orthosis, the sensor needs to be modeled accordingly. Even at this time, it can be easily modeled and manufactured with 3D printing. Tang et al. (2020) developed a 3D printed flexible pressure sensor for a smart insole using multi-walled carbon nanotube (MWCNT) [13] and Cao et al. (2022) proposed direct-ink-writing (DIW) 3D printed pressure sensor laminated graphene (below GR) [12]. Emon et al. (2019) offered a 3D printed multi-material soft pressure sensor composed of five stretchable layers. The two electrode layers between the top and bottom layers were formed using MWCNT/polymer composite. The pressure sensitive layer was formed between the two electrodes layer using ionic liquids [14]. In particular, studies applying pressure sensors to hand orthosis are as follows. Tan et al. (2020) prepared a 3D scanned hand and wrist orthosis composed of TPU and copper. On orthosis, conductive layers were 3D printed using carbon black TPU [15]. Wei et al. (2019) developed E-skin glove consisting of 15 strain sensors on the back and five pressure sensors on the back fingertips. Sensors sere DIW 3D printed using carbon nanoparticles [16]. Ntagios et al. (2020) reported that the sensors obtained are quite sensitive and can sense pressures as low as 1 kPa. The tightly integrated sensing within the 3D printed structures could pave the way for a new generation of truly smart material systems that can change their appearance and shape autonomously. The simplicity of the fabrication process presented here introduces a cost-effective alternative fabrication method for tactile sensing systems that otherwise require complex, expensive, and specialized equipment. Their potential for use as a sensor after imparting conductivity to the tip of the finger remains to be investigated [17].

In particular, additive manufacturing (AM) is used as a 3D method for manufacturing sensors or actuator. AM is a technique that combines materials to create physical objects specified in 3D model data. It is usually made by stacking several layers on top of each other [18]. AM processes can easily manufacture and offer great advantages in the time and cost [19,20]. In the AM technique, 3D printing parameters have a very important role in the physical properties of the printed samples. In particular, the infill pattern and infill density determine the internal structure and shape. According to adjust infill conditions, the output time and filament consumption can vary. Therefore, it can affect the printed output of weight, strength, flexibility, and so on [21,22,23,24].

Thus, this study aimed to confirm the most suitable 3D printing infill conditions and develop conductive 3D printed (below 3DP) fingertips for pressure sensors applied to the hand exoskeleton. The 3D printing infill conditions determine the internal structure and shape of the output and affect performance [21]. Thus, 3DP fingertips were fabricated applying three types of infill patterns and densities. Moreover, in particular, research was carried out on the index finger, which is most often used for hand gestures such as touching. First, the fingertip of index was prepared using 3D printed with TPU filament under various three types of infill patterns and three types of densities. Second, 3D printed fingertips were dip-coated with fabricated 8 wt% graphene/waterborne polyurethane composite solution. For the development of conductive fingertips which can sense the electrical touch, only the fingertips were coated with conductive materials. Third, manufactured 3DP fingertips were analyzed and their applicability confirmed in term of appearance property, weight changes, compressive property, and electrical property.

## 2. Materials and Methods

### 2.1. Materials

The TPU filament (eFlex, eSun, Shenzhen, China) is 1.75 mm in diameter with hardness of shore 87 A. The GR (GNP-UC, Carbon Nano Technology Co., Ltd., Pohang, Republic of Korea) and waterborne polyurethane (WPU, Akuarane 0264, T&L Co., Yong-in, Republic of Korea) were used to prepare the composite solution. The GR has 3–10 layers, length of 5–10 μm, and thickness of 3–6 nm. WPU has the solid proportion of 35 ± 1%, the ionic property of anion, and no co-solvent.

### 2.2. Preparation of 3DP Index Fingertips

**Figure 1:**
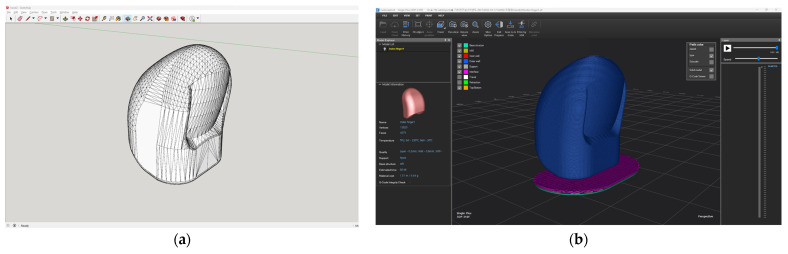
Modeling image of 3DP index fingertips: (**a**) Sketchup software; (**b**) Slicing software.

The 3D modeling of index fingertips was performed using Sketchup Go software (Trimble Inc., Westminster, CO, USA). In previous studies, hand modeling was performed with reference to the actual hand size. The fingertips were modeled separately to form a movable simulation model of hands. At the same time, connection modules are established to connect each knuckle. The three knuckles of the index finger, middle finger, ring finger, and little finger and the two knuckles of the thumb are modeled separately. Due to the complex structure of the hand, the linear tool in the software was used to grid the knuckle and the hand respectively. Finally, the ellipse tool, the rectangle tool, and the push-pull tool were used to build the connector [25]. The 3D modeling of fingertips saved in *.stl file. A .stl file was transformed into 3D printable .g-code format in slicing program (Cubicreator 4, Cubicon Co., Ltd., Seongnam, Republic of Korea). However, the current study used only index fingertips. Figure 1 shows modeling image of index fingertips. The size of index fingertip was as follows: 17.5 × 16.4 × 23.8 mm. The 3D printing conditions were set up as follows: layer height of 0.2 mm, top layer count of 5, bottom layer count of 3, nozzle temperature of 240 °C, bed temperature of room temperature, printing speed of 60 mm/s, build plate adhesion type of raft, 3 types of infill density of 20%, 50%, 80%, and infill patterns of Zigzag (below ZG), Triangle (below TR), and Honeycomb (below HN). Especially, the infill patterns and densities were chosen based on previous study [21]. The infill pattern was selected as each of the infill types, 2D for ZG, 3D for TR, and 3DF for HN. This approach was followed in consideration of output time and compressive property.

### 2.3. Preparation of Conductive GR/WPU Solution

The GR/WPU composite solution were prepared as follows. The GR content was selected through previous studies. In prior studies, the electrical properties were confirmed for each graphene content from 2 wt% to 16 wt%. As results, as the graphene content increased, the electrical property and electrical heating performance improved. However, in the case of 16 wt% graphene content, it was confirmed that it was brittle and crushed after annealing treatment [26,27,28,29]. Therefore, it proceeded with 8 wt% graphene content. 8 wt% of GR was added to 35 wt% of WPU solution dispersed in distilled water. The GR/WPU composite solution was stirred for 24 h using a digitally controlled hotplate stirrer (MSH-20D, Daihan Scientific, Daegu, Republic of Korea). The 8 wt% GR/WPU solutions were used after ultrasonic treatment for 1 h and stirring at 2400 rpm for 5 min with a rotator. 

### 2.4. Fabrication of Coated 3DP Index Fingertips with 8 wt% GR/WPU

The 3DP index fingertips were coated with 8 wt% GR/WPU solution by dip-coating. In the process of dip-coating, the samples were lifted and put into the GR/WPU solution every 10 s repeatedly for 6 times, so that the coating time of each sample is about 1 min. After the dip-coating, the coated samples were dried for 1 h in the oven of 60 °C.

### 2.5. Characterization

#### 2.5.1. Appearance Property

The appearance property of the 3DP index fingertip under various infill patterns and densities were analyzed comparatively between the slicing modeling and three types of the actual output. In the case of slicing modeling, the infill structure was confirmed with a solid model after applying the 3D printing conditions through the slicing program. As for the actual output, the 3DP index fingertip when the output progressed 50% and 100% and the coated 3DP index fingertip with 8 wt% GR/WPU composite solution were compared. In addition, the samples were weighted on automatic scales (PAG114, Ohaus Corp., Parsippany, NJ, USA). The difference in weight was analyzed by one-way ANOVA with the SPSS 27.0. All analyses of the significance level were set at *p* < 0.05. Moreover, the pick-up rate was calculated using Equation (1):

W (%) = {(W_c_ − W_0_)/W_0_} × 100 (1)

where *W* is pick-up rate, *W*_0_ is initial weight of 3DP index fingertips, and *W_c_* is weight of coated 3DP index fingertips with GR/WPU.

#### 2.5.2. Compressive Property

To confirm the compressive properties of 3DP index fingertip, it was measured according to the KS M ISO 604 standard. Experiments were implemented using a 5 kN load cell in the universal testing machine (AGS-X, Shimadzu Co., Kyoto, Japan). The samples were compressed in the -z direction and at a speed of 25 mm/min. The maximum strain was limited to 50% depends on the samples. Each sample was compressed three times and the obtained average value was used to analyze the compressive strength at 50% strain, initial modulus, and toughness. The difference in compressive properties was investigated by one-way ANOVA. All analyses of the significance level were set at *p* < 0.05.

#### 2.5.3. Electrical Property

Electrical properties of the 3DP index fingertip were analyzed using 2450 source meter (KEITHLEY, Washington, DC, USA). The voltage range was 0–30 V with interval 1 V. The current (A) was checked at applied voltage from 0 to 30 V. Relative resistance was checked under repeated compression conditions.

## 3. Results and Discussion

### 3.1. Appearance Property of 3DP Index Fingertips under Various Infill Patterns and Densities

**Figure 2:**
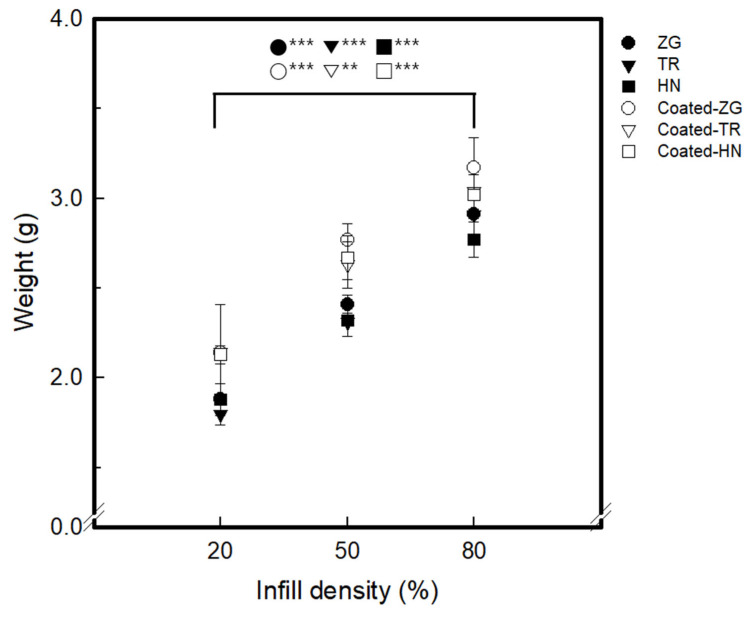
The weight of 3DP index fingertips and GR/WPU coated 3DP fingertips with various infill pattern and density (*p*-value: ** *p* < 0.01, *** *p* < 0.001).

**Figure 3:**
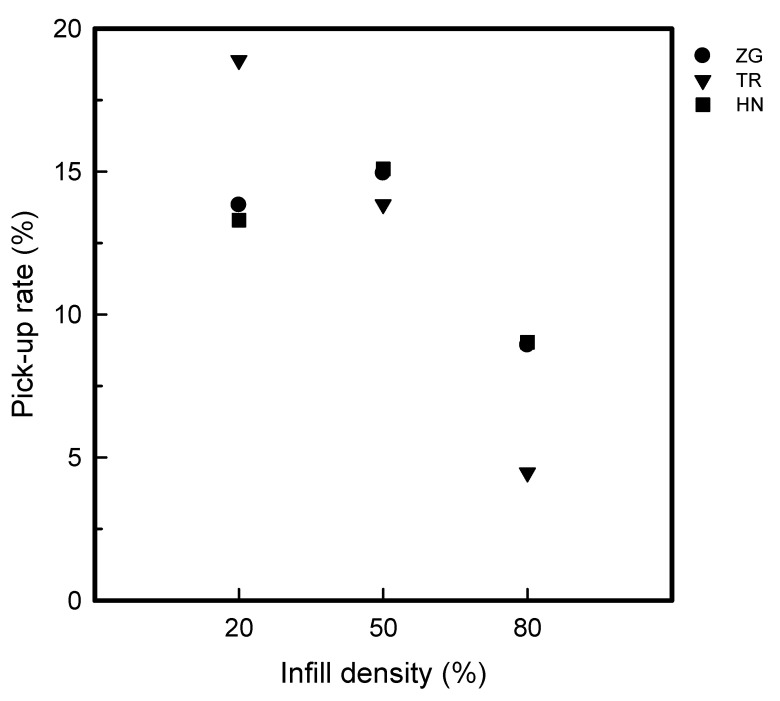
The pick-up rate of 3DP index fingertips with various infill patterns and densities.

Table 1 and Table 2 show the infill image and the image of 3DP index fingertips. Figure 2 and Figure 3 indicate weight and pick-up rate of 3DP index fingertips under various infill patterns and densities.

In the infill images, the internal structure was confirmed. As can be seen in Table 1, the infill density was found to be denser as it increased from 20% to 80%. A 50% progressed 3DP index fingertips also confirmed that internal structures were similarly manufactured compared to the slicing modeling. By infill patterns, ZG was deposited with unidirectional line layers crossing in two directions perpendicular to each other. It was confirmed that as the density increased, the number of units made by the vertically stacked two layers were increased more than three times. In the case of TR, the lines formed in three directions form one layer and the layers were stacked perpendicularly. At 20% infill density, it was confirmed that the layers were stacked with one central point and triangle unit can’t reveal because of the low density. At 50% and 80% infill density, the unit increased approximately three times. And HN was deposited with the same hexagonal layers. As same tendency, Similarly, the number of units increased by more than three times as the density increased. The 100% completed 3DP index fingertips were measured as 17.5 × 16.4 × 23.8 mm, the same as the input value. The actual printing time was as follows: As infill density increased, the actual time was 33, 35, and 37 min for ZG; 36, 39, and 42 min for TR, and 34, 38, and 43 min for HN.

The 100% completed 3DP index fingertips and coated 3DP index fingertips were analyzed by the weight. The weight of 3DP index fingertips by infill density increased as it increased from 20% to 80%. The weight of index 3DP fingertips was 1.80–1.88 g for 20% of infill density, 2.31–2.41 g for 50% infill density, and 2.77–2.91 g for 80% infill density. The weight of 3DP fingertips by infill pattern confirmed that ZG is the largest. As can be seen in the slicing image, this is confirmed because ZG has the highest layer density. As the number of TR units increased rapidly, the weight increase of TR was the largest. As the number of TR units increased rapidly, the weight increase of TR was the largest. In addition, TR was the lowest when the infill density was 20%, and HN was the lowest when the infill density was 50% and 80%. The weight of the coated 3DP index fingertips with 8 wt% GR/WPU gave the following results. The weight of coated 3DP index fingertips increases compared to initial 3DP fingertips. As for the infill density increasing, the weight of the coated 3DP fingertips by infill density also increased as the density increased. The weight was 2.13–2.14 g for 20% infill density, 2.63–2.77 g for 50% infill density, and 3.02–3.17 g for 80% infill density. The weight of 3DP fingertips by infill pattern showed the same tendency, with ZG being the largest in all infill density conditions. At 50% and 80% infill density, the weight of ZG was much higher than TR and HN. Therefore, one-way ANOVA was performed on the density-dependent variation of the infill pattern. A statistically significant difference of 3DP index fingertips and coated 3DP index fingertips was observed depending on the infill pattern in the analysis of variation by infill density (*p* < 0.01, *p* < 0.001). Moreover, it was confirmed that the initial F value was larger than that after coating. This was owing to resulted in sample weight differences after coating.

In addition, the weight difference within raw 3DP index fingertips and coated 3DP index fingertips was compared by pick-up rate. When compared by infill density, the value of pick-up rate was 13.30–18.89% for 20% infill density, 13.85–15.09% for 50% infill density, and 4.47–9.03% for 80% infill density. The value decreased as the infill density increased. This was considered because there was a lot of internal porous space at 20% infill density. A higher amount of 8 wt% GR/WPU composite solution could have penetrated to the samples. Moreover, it is considered that the TR at 20% infill density has a high pick-up rate as the inner density and unit counts was low.

### 3.2. Compressive Property of 3DP Finger Tips with Various Infill Pattern and Density

**Figure 4:**
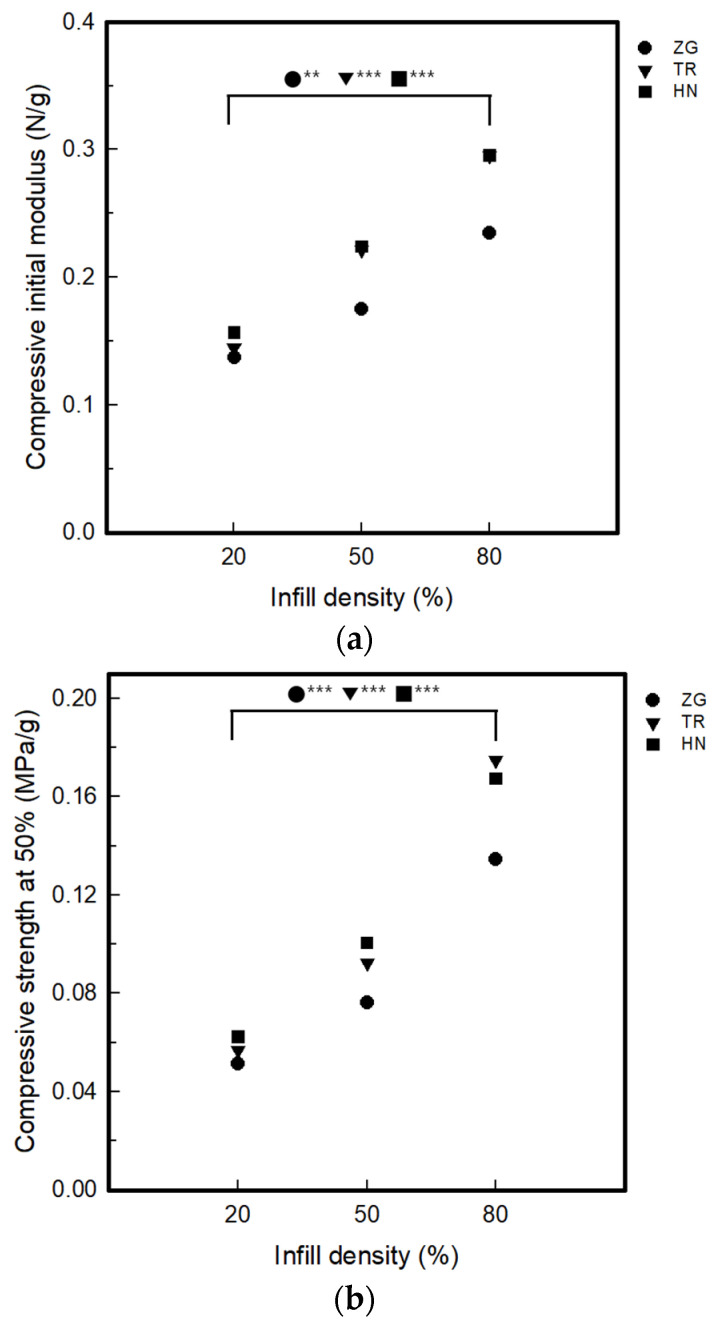

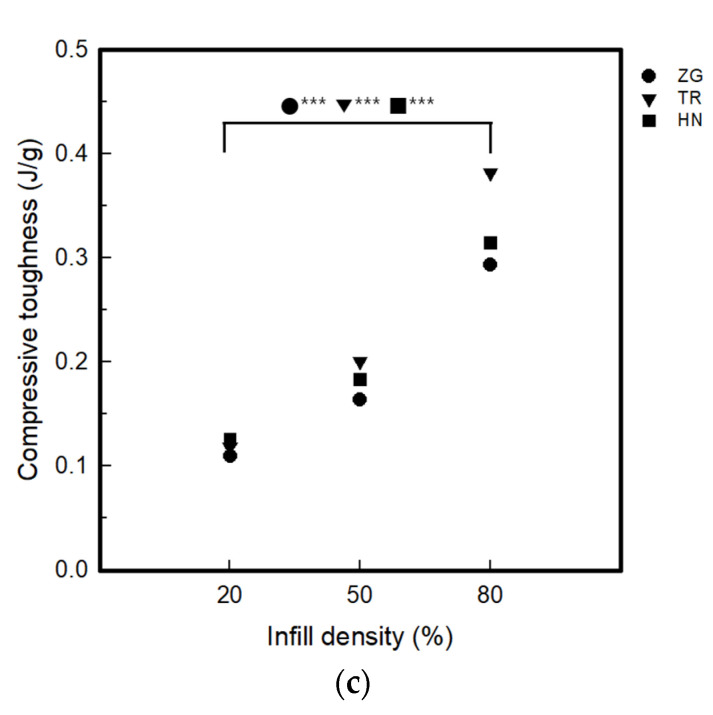
The compressive properties of the 3DP fingertips with various infill patterns and densities (*p*-value: ** *p* < 0.01, *** *p* < 0.001): (**a**) Compressive initial modulus; (**b**) Compressive strength at 50%; (**c**) Compressive toughness.

**Figure 5:**
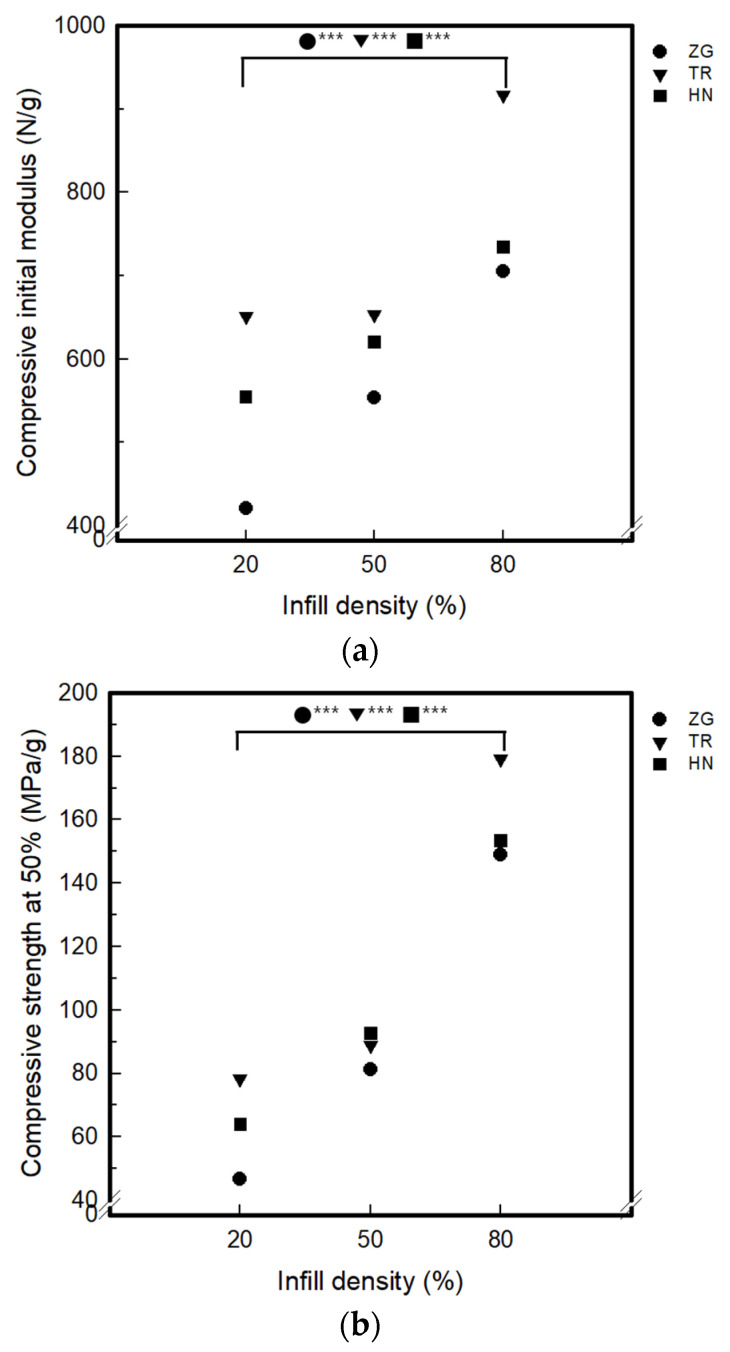

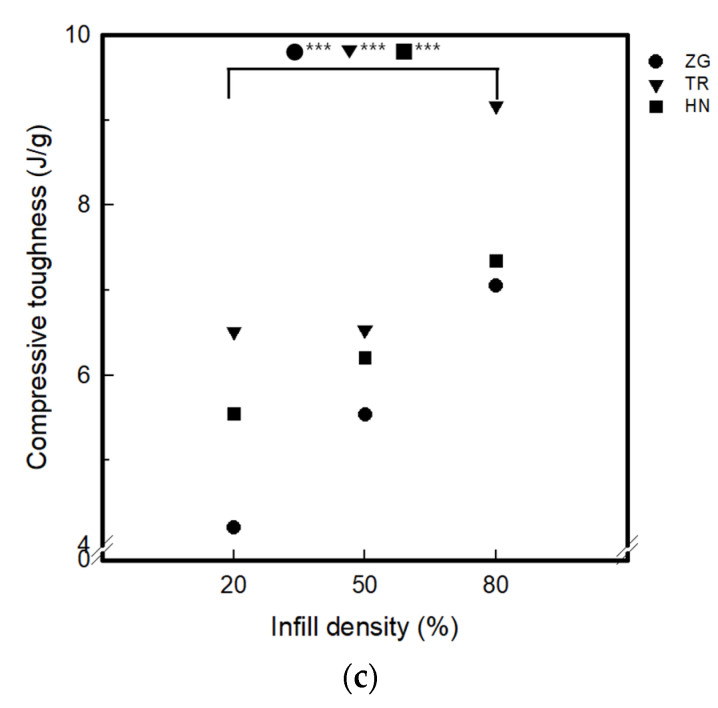
The compressive properties of the coated 3DP fingertips with various infill patterns and densities (*p*-value: *** *p* < 0.001): (**a**) Compressive initial modulus; (**b**) Compressive strength at 50%; (**c**) Compressive toughness.

Figure 4 and Figure 5 show the compressive properties of 3DP index fingertips and coated 3DP index fingertips with changing infill pattern and density. 

The compressive initial modulus of 3DP index fingertips was 0.14–0.16 N/g for 20% infill density, 0.17–0.22 N/g for 50% infill density, and 0.23–0.30 N/g for 80% infill density. Compressive initial modulus values for different infill densities increased with increasing densities from 20% to 80%. By infill pattern, at the infill density of 20% and 50%, it was showed that the highest HN value and the lowest ZG. On the other hand, when the infill density was 80%, the TR value was the largest and the ZG was the smallest. The compressive strength at 50% compression results of 3DP index fingertips are as follows. It was confirmed to be 0.05–0.06 MPa/g for 20% infill density, 0.07–0.10 MPa/g for 50% infill density, and 0.13–0.17 MPa/g for 80% infill density. The initial modulus and strength at 50% appeared to the same tendency. The compressive toughness of 3DP fingertips was 0.11–0.13 J/g for 20% infill density, 16–0.20 J/g for 50% infill density, and 0.29–0.38 J/g for 80% infill density. The strength increased as the density increased. By infill pattern, HN was the largest and ZG was the smallest at the infill density of 20%. On the other hand, at infill density of 50% and 80%, TR was the largest and ZG was the smallest. Therefore, in the effect of infill patterns, TR samples was higher than HN and ZG at 80% infill density. It can be seen that TR has the physical stability as the compressive initial modulus, strength and toughness of TR were the highest at infill density of 80%. Also, the toughness of TR pattern sample with 80% show the good and softness of samples with 20% infill density is good regardless of infill pattern. As can be seen in Table 1, in the case of TR, the layers were deposited in various directions other than ZG and HN. In the case of the AM process, the motion of the nozzle changes and the shape of the layer is determined according to the infill conditions. As the layers were stacked, a bonded portion between layers and a voided portion are created. Layer bonding can increase strength when an external force of compression or tensile is applied [30,31]. Thus, among the three infill patterns, TR which was deposited in the most diverse directions, had the most layer bonded parts. So, it was considered that the compressive toughness increased as the infill density increased.

As compressive properties result of the coated 3DP index fingertips are as follows. In case of compressive initial modulus, it appeared 421.06–650.66 N/g for 20% infill density, 553.87–652.97 N/g for 50% infill density, and 704.85–916.26 N/g for 80% infill density. The compressive strength at 50% compression was 46.66–78.14 MPa/g for 20% infill density, 81.23–92.76 MPa/g for 50% infill density, and 149.05–179.26 MPa/g for 80% infill density. The compressive toughness measured 4.21–6.51 J/g, 5.54–6.53 J/g, and 7.05–9.16 J/g relatively as infill density increased from 20% to 80%. Thus, as for the compressive properties, the sample became more rigid as the infill density increased. By each infill pattern, TR was the strongest and ZG was the softest, presenting a similar tendency to before coating. Moreover, the strength of coated 3DP index fingertips became stronger by approximately 1000 times than before coating. This is considered to be because coating with GR/WPU composite solution imparts mechanical strength [32]. The 8 wt% GR/WPU composite solution not only permeates into the interior, but also makes the exterior more rigid.

### 3.3. Electrical Property of 3DP Finger Tips with Various Infill Pattern and Density

**Figure 6:**
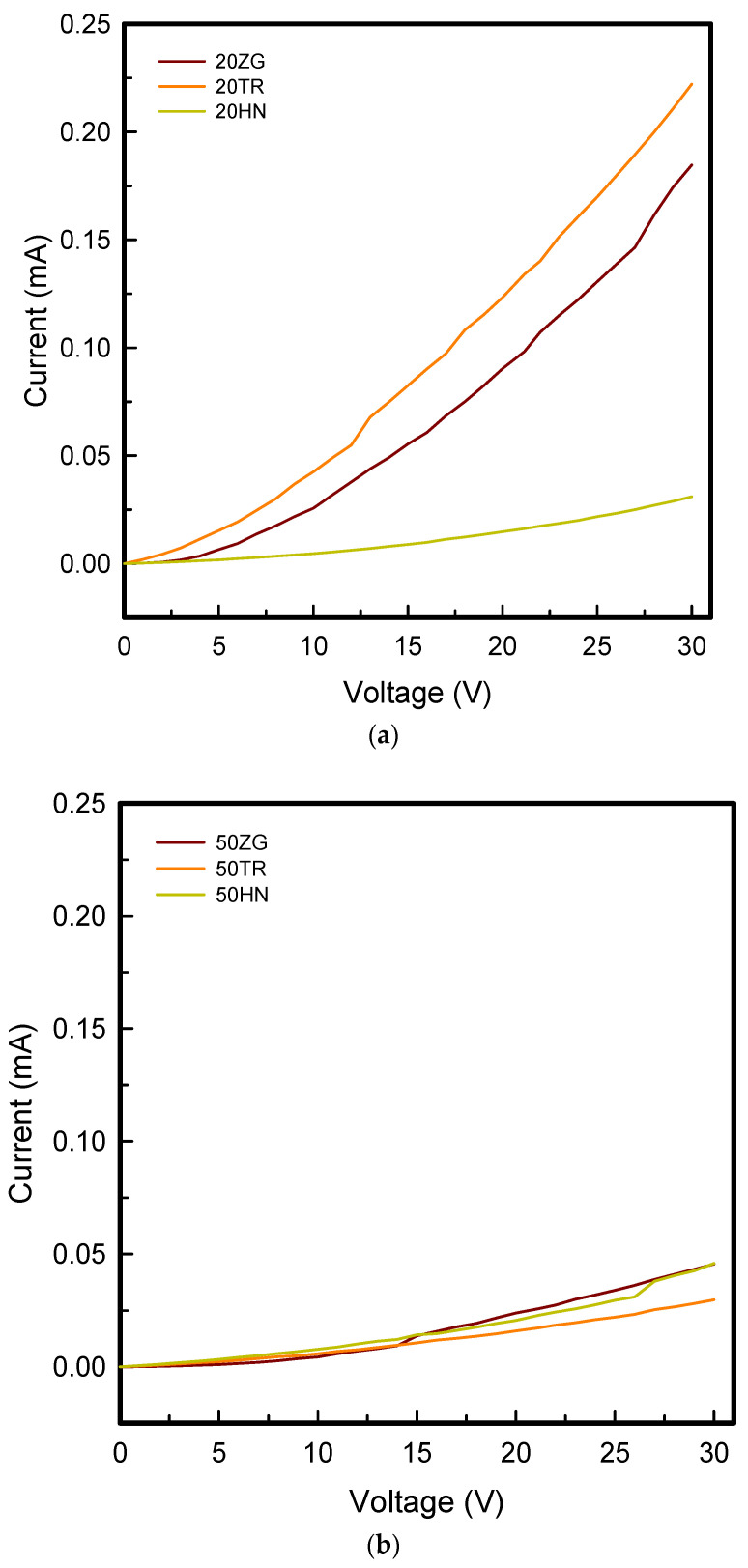

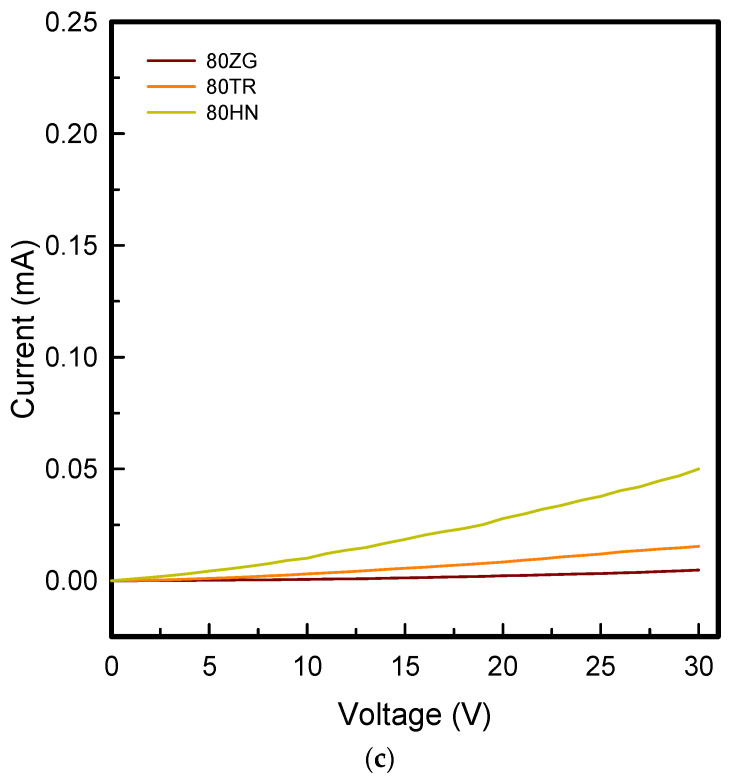
The current-voltage (I-V) curves of coated 3DP index fingertips with 8 wt% GR/WPU composite solution under various infill patterns and densities: (**a**) 20% infill density; (**b**) 50% infill density; (**c**) 80% infill density.

**Figure 7:**
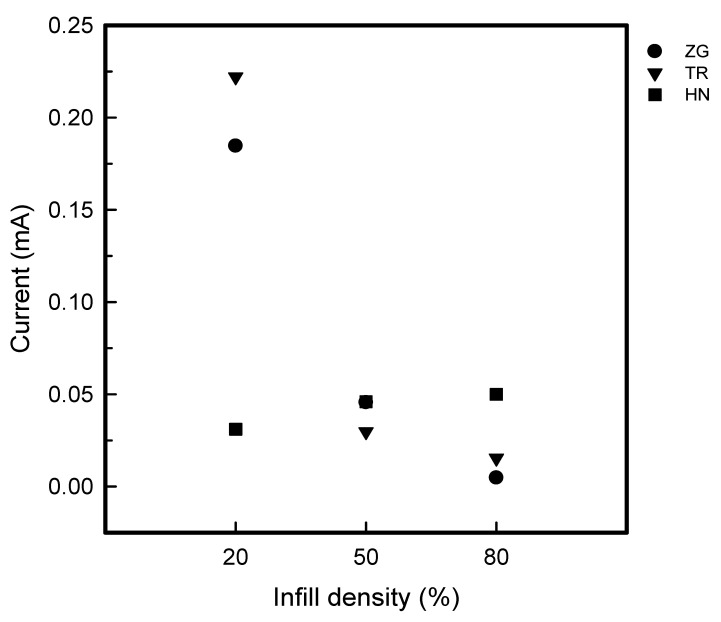
The current at 30V of coated 3DP index fingertips with 8 wt% GR/WPU composite solution under various infill patterns and densities.

**Figure 8:**
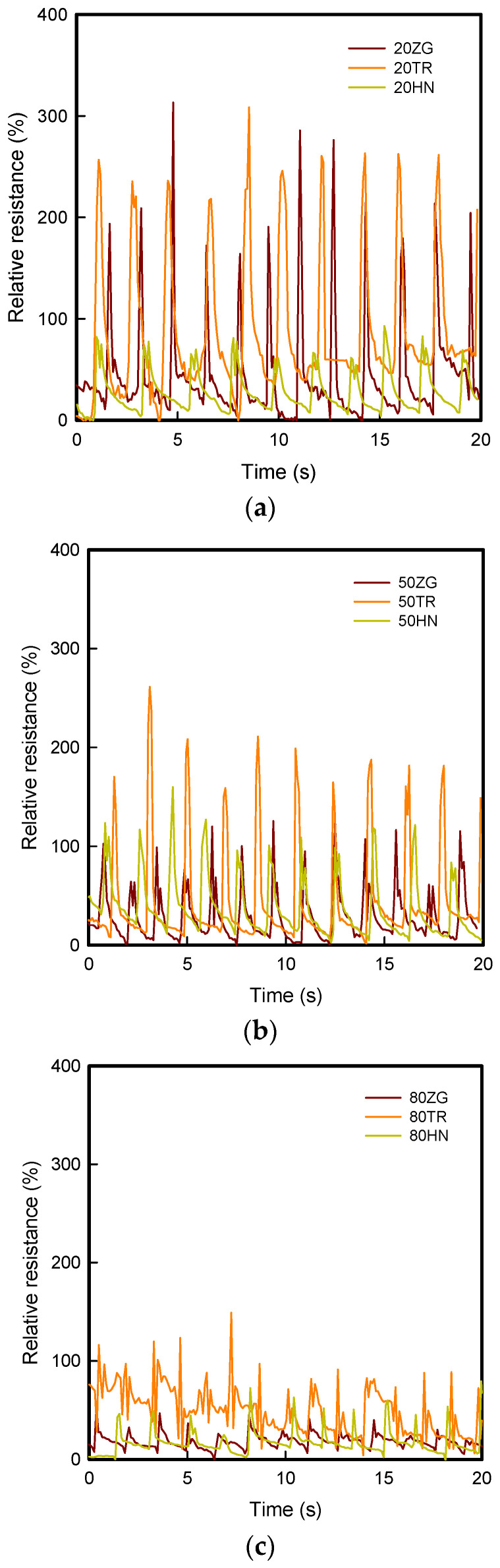
The relative resistance changes of coated 3DP index fingertips with 8 wt% GR/WPU composite solution under various infill patterns and densities: (**a**) 20% infill density; (**b**) 50% infill density; (**c**) 80% infill density.

Figure 6 and Figure 7 show current-voltage curves and current at 30 V of coated 3DP fingertips with 8 wt% GR/WPU under various infill pattern and density. Figure 8 presents the relative resistance changes of coated 3DP fingertips with 8 wt% GR/WPU under various infill patterns and densities.

As seen in Figure 5, the current was shown to be relatively maximum at 20% infill density and relatively small at 80% infill density. When the voltage was 30 V, in the range of 0.03–0.46 mA for 20% infill density, 0.02–0.05 mA for 50% infill density, 0.00–0.20 mA for 80% infill density were measured. Therefore, there was a tendency for the current to decrease as the infill density increases. This can be seen in relation to pick-up rate. The pick-up rate in Figure 3 also showed a tendency to decrease as the infill density increased from 20% to 80%. It can be seen that the 8 wt% GR/WPU solution was dip-coated with a infill density of 20%, which has the highest internal porous space, and the highest amount of solution was coated. Also, it was confirmed that the electrical properties were excellent at infill density of 20%, which had the highest pick-up rate. Moreover, TR was the best at 20% infill density, while HN was superior at 80% in current. The internal structure is relatively simple at 20% infill density. So, it can be considered that TR which layers were stacked in the most diverse directions was excellent. On the other hand, at high infill density of 50% and 80%, HN deposited identical layers had the best electrical properties.

The results of relative resistance in Figure 8 are as follows. In case of 3DP index fingertips with 20% infill density, the relative resistance shows the range of 300%. It was previously confirmed that the current increases as the infill density increases in the same tendency as the current. In addition, in case of samples with various infill patterns, the relative resistance of the TR sample was larger than that of HN. It was judged that the electrical properties by compression are excellent.

## 4. Conclusions

This study intended to develop a conductive pressure sensor applied to the hand exoskeleton and to confirm potential of manufactured conductivity 3DP fingertips to sense finger touching. For preparing coated 3DP fingertips, fingertips were 3D printed with TPU filament under three types of infill patterns and densities. An additional fingertip was dip-coated with 8 wt% GR/WPU composite solution. Then, the appearance property, weight changes, compressive property, and electrical property were evaluated for confirming the usability of coated 3DP fingertips.

As a result, the weight of 3DP fingertips increased by infill density increased. By infill pattern, ZG was largest. While TR was the lowest at the infill density 20% and HN was the lowest at the infill density 50% and 80%. After coating with 8 wt% GR/WPU composite solution, the weight of coated 3DP fingertips increased. The pick-up rate decreased as the infill density increased. It can be considered that a significant amount of composite solution could have penetrated because of the internal porous space at 20% infill density. In the case of compressive properties, it was confirmed that compressive strength increased as infill density increased. According to infill patterns, TR has greater physical stability than HN and ZG as an internal layer structure deposited in various directions. TR was deposited in the most diverse directions, so it had the most layer bonded parts. In particular, coated 3DP index fingertips improved the compressive properties of TR at all infill densities. The electrical properties presented a tendency to decrease as the infill density increased. These results are in accordance with the pick-up rate. Moreover, according to infill patterns, TR for 20% infill density and HN for 50% and 80% had the best conductivity. TR stacked in the most diverse directions was excellent at 20% infill density for a relatively simple structure. In addition, conductivity by compression was also larger for the TR.

Therefore, in this study, we confirmed the possibility of the conductivity 3DP fingertips for a pressure sensor. In addition, the infill pattern of TR with 20% infill density was most suitable for use as a conductive compression sensor condition. Additionally, in the case of 20% infill density, it presented softness and elasticity, in addition to saving time and money. In future research, a robotic exoskeleton for hand-rehabilitation will be developed based on the research results. Furthermore, a manufactured conductive filament synthesized with a conductive polymer and thermoplastic polyurethane will be applied to the 3DP pressure sensor, strain sensor, and so on.

## Figures and Tables

**Table 1:** Infill image of 3DP fingertips of index under various infill patterns and densities.

Infill Density (%)	Infill pattern					
	ZG		TR		HN	
	Slicing Model	Progressed 50%	Slicing Model	Progressed 50%	Slicing Model	Progressed 50%
20	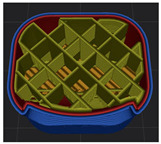	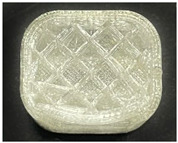	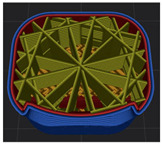	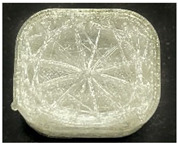	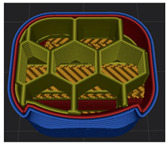	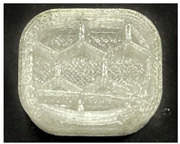
50	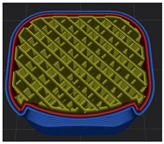	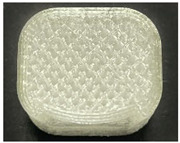	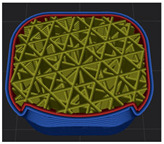	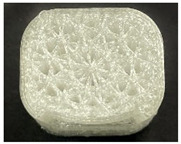	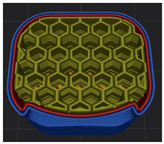	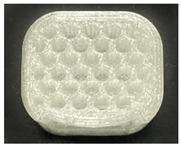
80	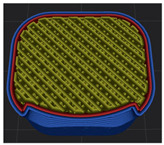	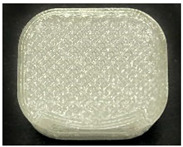	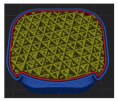	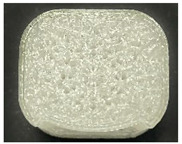	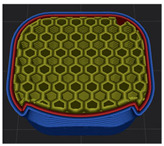	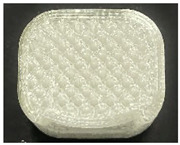

**Table 2:** Sample images of 3DP fingertips and coated 3DP index fingertips under various infill patterns and densities.

Infill Pattern	Infill Density (%)		Initial			Coated		
			Front	Side	Back	Front	Side	Back
ZG	20	Image	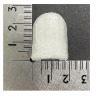	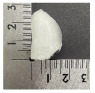	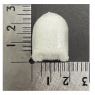	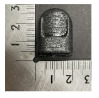	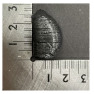	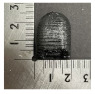
		Weight (g)	1.88 ± 0.03			2.14 ± 0.04		
	50	Image	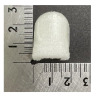	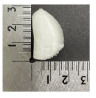	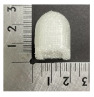	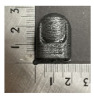	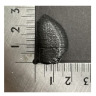	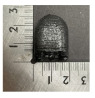
		Weight (g)	2.41 ± 0.05			2.77 ± 0.09		
	80	Image	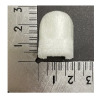	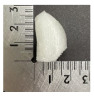	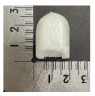	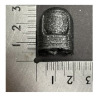	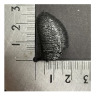	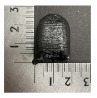
		Weight (g)	2.91 ± 0.14			3.17 ± 0.17		
TR	20	Image	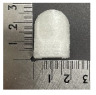	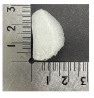	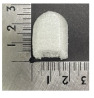	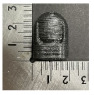	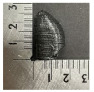	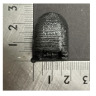
		Weight (g)	1.80 ± 0.06			2.14 ± 0.27		
	50	Image	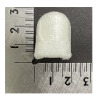	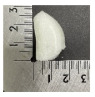	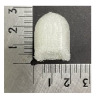	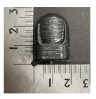	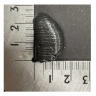	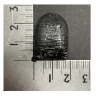
		Weight (g)	2.31 ± 0.01			2.63 ± 0.13		
	80	Image	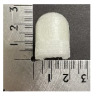	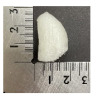	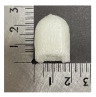	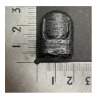	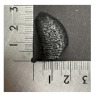	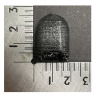
		Weight (g)	2.91 ± 0.13			3.04 ± 0.01		
HN	20	Image	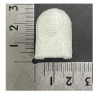	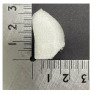	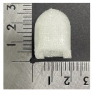	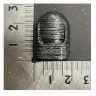	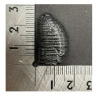	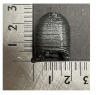
		Weight (g)	1.88 ± 0.09			2.13 ± 0.05		
	50	Image	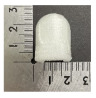	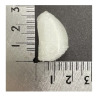	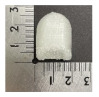	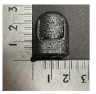	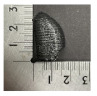	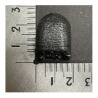
		Weight (g)	2.32 ± 0.09			2.67 ± 0.12		
	80	Image	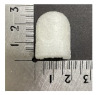	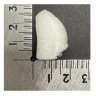	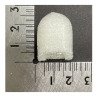	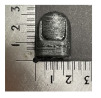	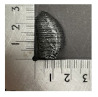	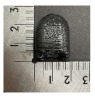
		Weight (g)	2.77 ± 0.10			3.02 ± 0.11		

## Data Availability

Not applicable.
